# First‐In‐Human Dose Finding Study of Venadaparib (IDX‐1197), a Potent and Selective PARP Inhibitor, in Patients With Advanced Solid Tumors

**DOI:** 10.1002/cam4.70576

**Published:** 2025-02-13

**Authors:** Sung‐Bae Kim, Kyun‐Seop Bae, Jae Lyun Lee, Won Sik Lee, Chan‐Young Ock, Myong‐Jae Lee, Jeongsook Bang, Min Ju Hong, Eun‐Jihn Roh, Kyoung Soo Ha, Jong‐Ha Lim, Yong‐Man Kim

**Affiliations:** ^1^ Department of Oncology, Asan Medical Center University of Ulsan College of Medicine Seoul Republic of Korea; ^2^ Department of Clinical Pharmacology & Therapeutics, Asan Medical Center University of Ulsan College of Medicine Seoul Republic of Korea; ^3^ Idience Co. Ltd. Seoul Republic of Korea; ^4^ Idience Inc. Irvine California USA; ^5^ Ildong Pharmaceutical Co. Ltd Gyeonggi‐do Republic of Korea; ^6^ Department of Obstetrics and Gynecology, Asan Medical Center University of Ulsan College of Medicine Seoul Republic of Korea

**Keywords:** BRCA mutation, PAR inhibitory effect, PARP inhibitor, venadaparib

## Abstract

**Background:**

Venadaparib, a novel poly (ADP‐ribose) polymerase (PARP) inhibitor, has demonstrated high PARP‐1/2 selectivity over other PARP family members and exhibited strong PARP‐trapping activity, effectively inhibiting tumor growth in homologous recombination deficient (HRD) cancer in vitro and in vivo.

**Methods:**

This phase 1, dose‐finding study evaluated the safety, tolerability, pharmacokinetics, pharmacodynamics and anticancer efficacy of venadaparib as monotherapy in patients with advanced solid tumors that progressed after standard‐of‐care therapy. The study employed a conventional 3+3 design, with doses ranging from 2 mg/d to 240 mg/d.

**Results:**

Among the 32 enrolled patients, the most common tumor types were breast (16 patients) and ovarian (12 patients) cancers. No dose‐limiting toxicities (DLTs) were observed up to 240 mg/d. The most frequent grade 3 or 4 adverse events were anemia (50%), neutropenia (22%) and thrombocytopenia (6%). Tumor shrinkage by Response Evaluation Criteria in Solid Tumours (RECIST) was observed at doses ≥ 40 mg/d, regardless of BRCA mutation status.Two partial responses out of four ovarian cancer patients receiving venadaparib ≥ 40 mg/d were reported. Clinical benefit, defined as stable disease or partial response, was observed at the lowest tested dose. Venadaparib exhibited ≥ 90% PAR inhibitory effect in pharmacodynamic analysis from 10 mg/d based on tumor samples. The recommended phase 2 dose (RP2D) was defined as 160 mg once daily.

**Conclusions:**

Further studies are warranted to explore efficacy and safety of venadaparib in other tumor types and in combination with various agents, as well as to explore relevant biomarkers. (ClinicalTrials.gov ID: NCT03317743).

## Graphical abstract

In this phase 1, dose escalating study, venadaparib (IDX‐1197) had acceptable safety profile with wide therapeutic window with no DLT observed. Clinical pharmacology assessment demonstrated favorable pharmacokinetic characteristics for once daily dosing and pharmacodynamic PAR inhibition from lower doses aligned with signal of efficacy in heavily treated patients with BRCA mutations. These warrants further investigation as monotherapy and in combination.

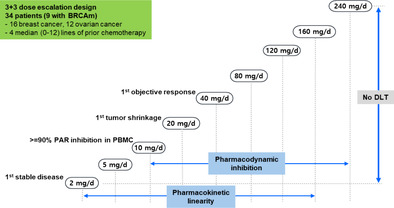

## 1 Introduction

The poly (ADP‐ribose) polymerase (PARP) is a ubiquitous nuclear enzyme involved in DNA repair. It catalyzes the transfer of ADP‐ribose to target proteins (PARylation) by utilizing nicotinamide adenine dinucleotide (NAD+) [1]. Among the 17 PARP family members, PARP‐1 and PARP‐2 are involved in DNA damage repair and are essential for cell survival [2]. Many proteins are involved in the DNA damage response (DDR), which can be affected by alterations in those proteins. Inhibition of PARP‐1 leads to inadequate function of the DDR repair system and inhibits replication in breast cancer genes 1 and 2 (BRCA1/2) protein‐deficient cancer cells, a concept known as “synthetic lethality” [3, 4]. Based on this theory, PARP inhibitor treatment has exhibited therapeutic efficacy in patients with homologous recombination deficiency (HRD) [5]. PARP inhibitors have demonstrated an improved toxicity profile, efficacy, and overall survival (OS) in treating HRD‐associated solid cancers [6, 7, 8]. To date, four PARP inhibitors have been approved by the US Food and Drug Administration (FDA): Lynparza in 2014 (AstraZeneca), Rubraca in 2016 (Clovis Oncology Inc.), Zejula in 2017 (Tesaro Inc.), and Talzenna in 2018 (Pfizer) [9, 10, 11, 12]. However, PARP inhibitor monotherapy has low anticancer activity against wild type (wt) cancers without BRCA mutations, especially platinum‐resistant or homologous recombination repair (HRR)‐proficient cancers [13]. Therefore, studies on the use of PARP inhibitors in combination with other chemotherapy have been under active investigation in various cancer types but has faced challenges due to hematologic or gastrointestinal toxicities associated with the inhibition of other PARP family enzyme such as PARP5 or PARP6 is related with these [14]. Therefore, research and development to improve the sensitivity and safety profile of PARP inhibitors are ongoing [15, 16, 17]. Also, for the effective treatment of cancers with HRD, developing promising treatment strategies and options that circumvents PARPi resistance is still in great demand [18].

Venadaparib (also known as IDX‐1197 or NOV1401) is an orally administered small molecule PARP inhibitor with bicyclic lactam amide structure. Venadaparib shows high PARP‐1/2 selectivity over other PARP family members and exhibits strong PARP‐trapping activity at the DNA single‐strand cleavage site, thereby effectively inhibiting the mechanism of cancer cell DNA damage recovery [19]. In in vitro testing, venadaparib exhibited superior inhibitory activity against BRCA‐mutated cancer cell lines compared to other PARP inhibitors and high specificity in inhibiting the growth of HR‐deficient cancer cells [19, 20]. Also, BRCA‐deficient cells were more sensitive to venadaparib than wild‐type cells, and tumor growth inhibition was also demonstrated in BRCA‐deficient ovarian, breast, and pancreatic cancer xenografts [21].

Previously, we have shown that venadaparib has drug‐able physicochemical properties with a favorable pharmacologic profile. Here, we describe a clinical evaluation of this novel orally active PARP inhibitor.

## 2 Methods/Experimental

### 2.1 Patients

The study was performed at the Asan Medical Center, Seoul, Republic of Korea. Eligibility criteria were: age 19 years or older; diagnosed with advanced solid tumors that progressed after standard‐of‐care treatment with no effective therapy; no prior PARP inhibitor treatment; able to swallow the investigational product and continue receiving study treatment; life expectancy ≥ 12 weeks at enrollment; written consent to study participation, and Eastern Cooperative Oncology Group (ECOG) performance status of 2 or less. The last laboratory test results obtained within 14 days prior to study enrollment had to indicate adequate hematologic, hepatic, and renal function. Women of childbearing potential must agree to use acceptable methods of contraception during the study.

Patients were excluded if they have history of hypersensitivity reactions to any of the components of the investigational product or other drugs of the same class, symptomatic or uncontrolled central nervous system disease, or a history of myelodysplastic syndrome (MDS) or pre‐treatment cytogenetic test results indicative of the risk of MDS or acute myelocytic leukemia.

### 2.2 Study Design

Venadaparib was given at 2, 5, 10, and 20 mg, once daily, for 2 weeks on and 1 week off of every 3 weeks, (Cohort 1–4 respectively); then increased to 20, 40, 80, 120, 160, 240 mg, once daily, given continuously in 3‐week cycles (Cohort 5–10 respectively) in reference to the phase I trial of Olaparib for close monitoring of safety [22]. Based on ICH S9 guidelines, 2 mg/d was selected as the starting dose, considering the highest non‐severely toxic dose (HNSTD) of 0.3 mg/kg/day in a toxicity test using beagle dogs [23].

Dose escalation was performed based on a conventional 3 + 3 dose‐escalation design, starting from the lowest dose level (2 mg/d) until the maximum tolerable dose (MTD) was determined. Dose‐limiting toxicities (DLTs) and pharmacokinetics were assessed during the first cycle. This was a phase 1 trial, with additional objectives to determine safety, pharmacokinetic, pharmacodynamic and efficacy profiles, based on which the recommended phase 2 dose (RP2D) would be determined.

### 2.3 Study Assessment

Safety evaluations were conducted at baseline and at weekly visits thereafter. Each evaluation consisted of a history and physical examination; laboratory panels, including a complete blood count, levels of clotting factors and electrolytes, liver‐ and renal‐function tests; and an electrocardiographic recording. Adverse events were graded according to the Common Terminology Criteria for Adverse Events (version 4.03) [24].

Pharmacokinetic and pharmacodynamic studies were performed during the first cycle of treatment. Plasma samples were analyzed for venadaparib concentration using liquid‐liquid extraction followed by high‐performance liquid chromatography, with detection by mass spectrometry. The plasma concentration–time data were analyzed using noncompartmental analysis (WinNonLin, version 8.0, SAS version 9.4 and R version 3.6.3) to derive pharmacokinetic parameters after the first dose (single‐dose parameters) and after the dose on Day 14 (multiple‐dose parameters). Pharmacodynamic studies were performed by evaluating Poly (ADP‐ribose) in peripheral blood mononuclear cells and quantifying tumor‐tissue lysates using the HT PARP in vivo Pharmacodynamic ELISA Kit II (R&D Systems, 4520‐096‐K) [25, 26, 27, 28].

Radiologic assessments of computed tomography or magnetic resonance imaging were carried out every 2 cycles and graded according to the Response Evaluation Criteria in Solid Tumors (RECIST version 1.1) [29].

Available archival samples were analyzed retrospectively for genetic information; tumor tissue was analyzed by Myriad myChoice HRD Plus CDx Test, Myriad genetics Inc., Salt Lake City, UT, USA; peripheral blood mononuclear cell (PBMC) NGS of BRCA gene was performed using Axen BRCA1/2 Cancer panel, Macrogen Inc., Seoul, Korea; plasma ctDNA analysis of HRD gene was implemented by Guardant OMNI panel, Guardant Health, Redwood City, CA, USA [30, 31, 32].

## 3 Results

### 3.1 Study Patients

**FIGURE 1:**
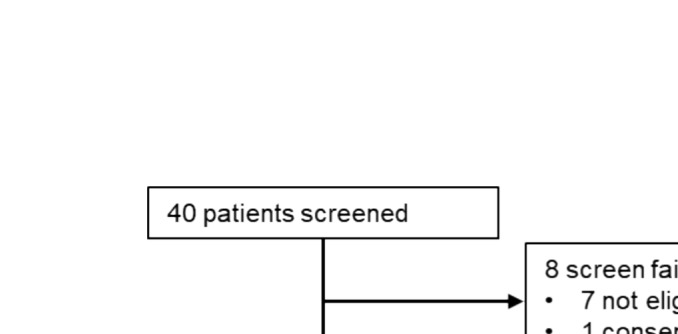
Patient disposition.

Forty patients were screened and 32 patients with histologically or cytologically confirmed advanced solid tumors were enrolled from November 2017 to June 2020. (Figure 1) All patients had received at least one prior chemotherapy regimen except for one ovarian cancer patient (patient 028) who had received adjuvant chemotherapy but no palliative chemotherapy. No patients had been treated with PARP inhibitors. Their baseline characteristics are presented in Table 1. Each dose group enrolled three patients except for the 20 and 160 mg/d groups due to disease progression and visit non‐compliance during the first cycle for DLT assessment, respectively. Descriptions of the evaluated venadaparib doses in 10 separate cohorts are provided in Table S1 in Appendix S1. Sixteen breast cancer (8 hormone‐receptor positive (HR+), 4 triple‐negative (TNBC), 4 epidermal growth receptor 2 (HER2) positive) and 12 ovarian cancer patients were enrolled. Based on medical history, nine patients (3 ovarian, 6 breast cancer) had pathogenic BRCA mutation (3 germline (g)BRCA1, 1 somatic (s)BRCA1, 5 gBRCA2), while 15 patients (6 ovarian, 5 breast, 2 uterine, 1 endometrial, 1 prostate cancer) had wild‐type BRCA and 3 patients (2 ovarian, 1 breast) had wild‐type HRD. Eleven of 15 BRCA wild‐type and 3 HRD wild‐type were confirmed by archival sample analysis. (Table S1) Most patients were heavily treated before enrollment; they had a median of 4 (range 0–12) prior lines of palliative chemotherapies; 62.5% of breast cancer patients had 3 or more prior palliative chemotherapies; ovarian cancer patients (including 1 platinum naïve patient) had a median of 2 (range 0–4) prior platinum‐containing chemotherapy regimens (Table S1).

**TABLE 1:** Baseline demographics and disease characteristics.

Characteristics	*N* = 32
Median age, years (range)	54.5 (31–75)
Male, *n* (%)	1 (3.1)
ECOG performance status, *n* (%)	
0	6 (19.0)
1	26 (81.0)
Tumor type, *n* (%)	
Breast cancer	16 (50.0)
Hormone‐receptor positive	8 (25.0)
Triple‐negative	4 (12.5)
Human epidermal growth factor receptor 2 (HER2) positive	4 (12.5)
Ovarian cancer	12 (37.5)
Platinum resistant	9 (9.4)
Platinum sensitive	3 (3.1)
Uterus	2 (6.3)
Endometrium	1 (3.1)
Prostate	1 (3.1)
Mutation status, *n* (%)	
Germline BRCA1	3 (9.4)
Somatic BRCA1	1 (3.1)
Germline BRCA2	5 (15.6)
Somatic BRCA2	0
BRCA1 and 2	0
Median prior palliative chemotherapy, *n* (range)	4 (0–12)
Breast cancer	3 (1–8)
Ovarian cancer	5 (0–9)
Median prior platinum regimen of ovarian cancer, *n* (range)	2 (0–4)

### 3.2 Safety

Safety was assessed in a total of 32 subjects who received at least one dose of the investigational product and had safety evaluated at least once. The maximum tolerated dose (MTD) was not determined because no DLT was observed during the Cycle 1, the DLT assessment period, across all dose levels from 2 to 240 mg/d.

Table 2 presents the common toxicities (≥ 5% incidence) possibly related to the study drug, which were largely grade 1 or 2 and included anemia (59%), neutropenia, (28%) nausea (38%), vomiting (16%), decreased appetite (16%), dyspepsia (13%), and fatigue (16%). The most frequent Grade 3 or 4 adverse reactions were anemia (50%), and neutropenia (22%).

**TABLE 2:** Venadaparib‐related adverse event found at least 5%.

Adverse events	All grades (*n* = 32)	Grade 3–4 (*n* = 32)
Subject with any AEs	31 (97%)	18 (56%)
Blood and lymphatic system disorders		
Anemia	19 (59%)	16 (50%)
Neutrophil count decreased	9 (28%)	7 (22%)
Platelet count decreased	2 (6%)	2 (6%)
Gastrointestinal disorders		
Nausea	12 (38%)	
Vomiting	5 (16%)	
Dyspepsia	4 (13%)	
Abdominal pain	2 (6%)	
Metabolism and nutrition disorders		
Decreased appetite	5 (16%)	1 (3%)
Hypoalbuminaemia	2 (6%)	1 (3%)
Hypertriglyceridaemia	2 (6%)	
General disorders and administration site conditions		
Fatigue	5 (16%)	
Asthenia	2 (6%)	1 (3%)
Influenza like illness	2 (6%)	
Nervous system disorders		
Headache	3 (9%)	
Infections and infestations		
Urinary tract infection	2 (6%)	

Of the 32 patients, 4 (12.5%) reported at least one dose reduction, most of whom (3 patients) had reductions due to an adverse event (AE) such as anemia, thrombocytopenia, or neutropenia. Anemia and thrombocytopenia in one patient in the 20 mg/d continuous dosing group (Cohort 5) and ileus in one patient receiving 20 mg/d for 2 of every 3 weeks (Cohort 4) led to permanent withdrawal.

There were no adverse reactions leading to death. No significant increase in the frequency or grade of adverse effects was observed in BRCA1 or BRCA2 mutation carriers compared to non‐carriers. (Table S4).

### 3.3 Pharmacokinetic Study

**FIGURE 2:**
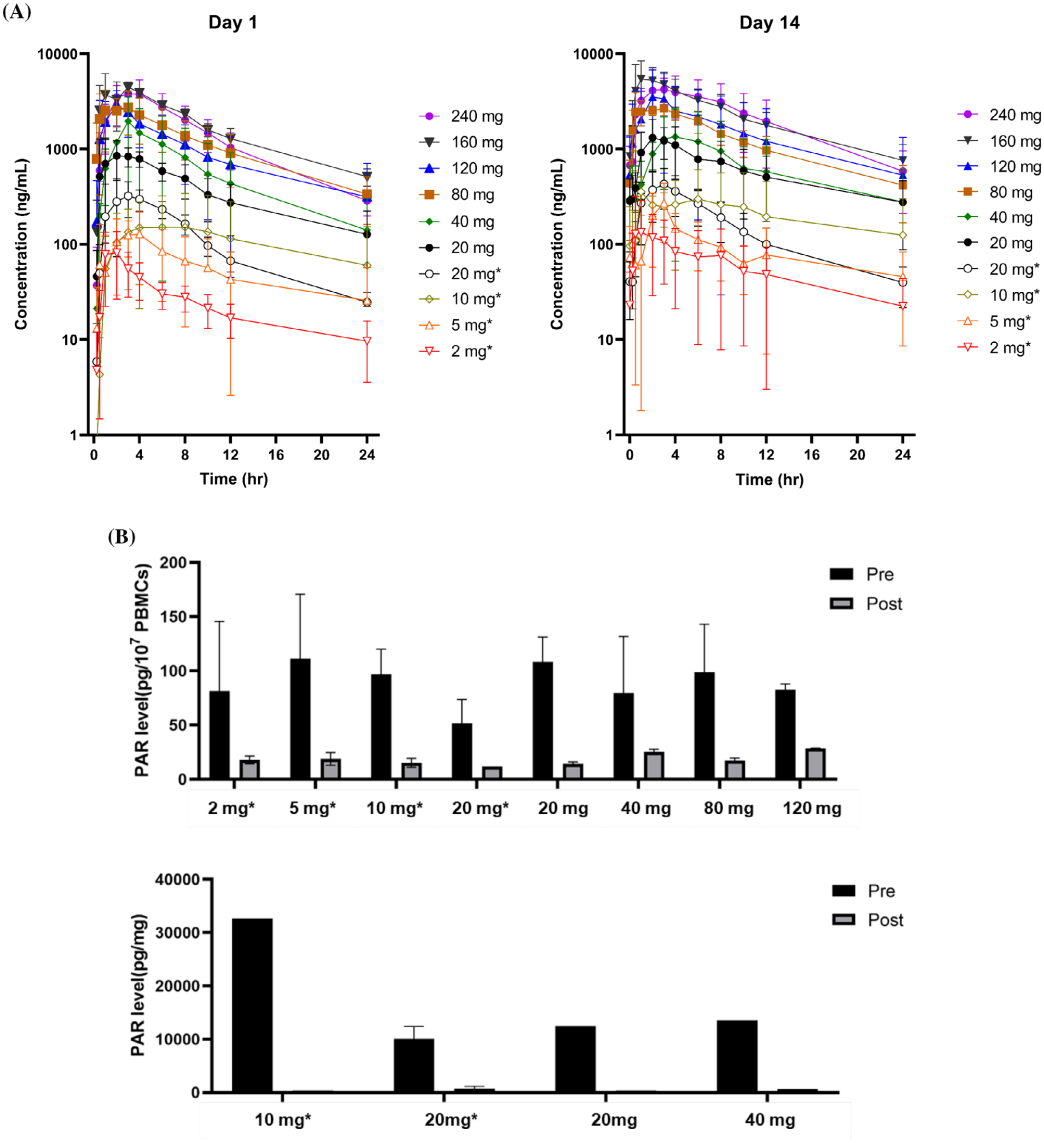
(A) Plasma concentration‐time curves of venadaparib on Day 1 and Day 14. *2 week on 1 week off, otherwise continuously administered. (B) Poly (ADP‐ribose) (PAR) level pre‐and 24 h post‐dose from peripheral‐blood mononuclear cells (upper) and tumor‐tissue cell lysates (lower) *Venadaparib administered 2 week on, 1 week off.

Results of pharmacokinetic studies indicated that venadaparib is rapidly absorbed, with the maximum plasma concentration (C_max_) observed between 1 and 3 h after dosing (Figure 2A). Thereafter, plasma concentrations declined biphasically, with a half‐life of approximately 8.7 h (Tables S2,S3). The mean volume of distribution was 75 L, and the mean plasma clearance rate was 6.3 L per hour. After 14 days of daily administration, drug exposure increased by an average of 99%; there was no marked dose‐dependency in the pharmacokinetics of venadaparib (Figure 2A). Notably, C_max_ and area under the time‐concentration curve (AUC) showed dose independency up to 160 mg/d (Cohort 9), where these values reached their maximum.

### 3.4 Pharmacodynamics

PARP inhibition was evidenced by the loss of signal from PAR (a biomarker for PARP activity) after treatment. Peripheral blood mononuclear cell (PBMC) analysis results showed that venadaparib administration from 2 to 120 mg once daily achieved a persistent PAR inhibitory effect up to 24 h.

Inhibition of PARP by more than 90%, compared to baseline, was observed in cells from patients treated with 10 mg or higher of venadaparib daily. Significant PARP inhibition was also confirmed in tissue biopsies from patients receiving 10, 20 and 40 mg once daily (Figure 2B).

### 3.5 Efficacy

**FIGURE 3:**
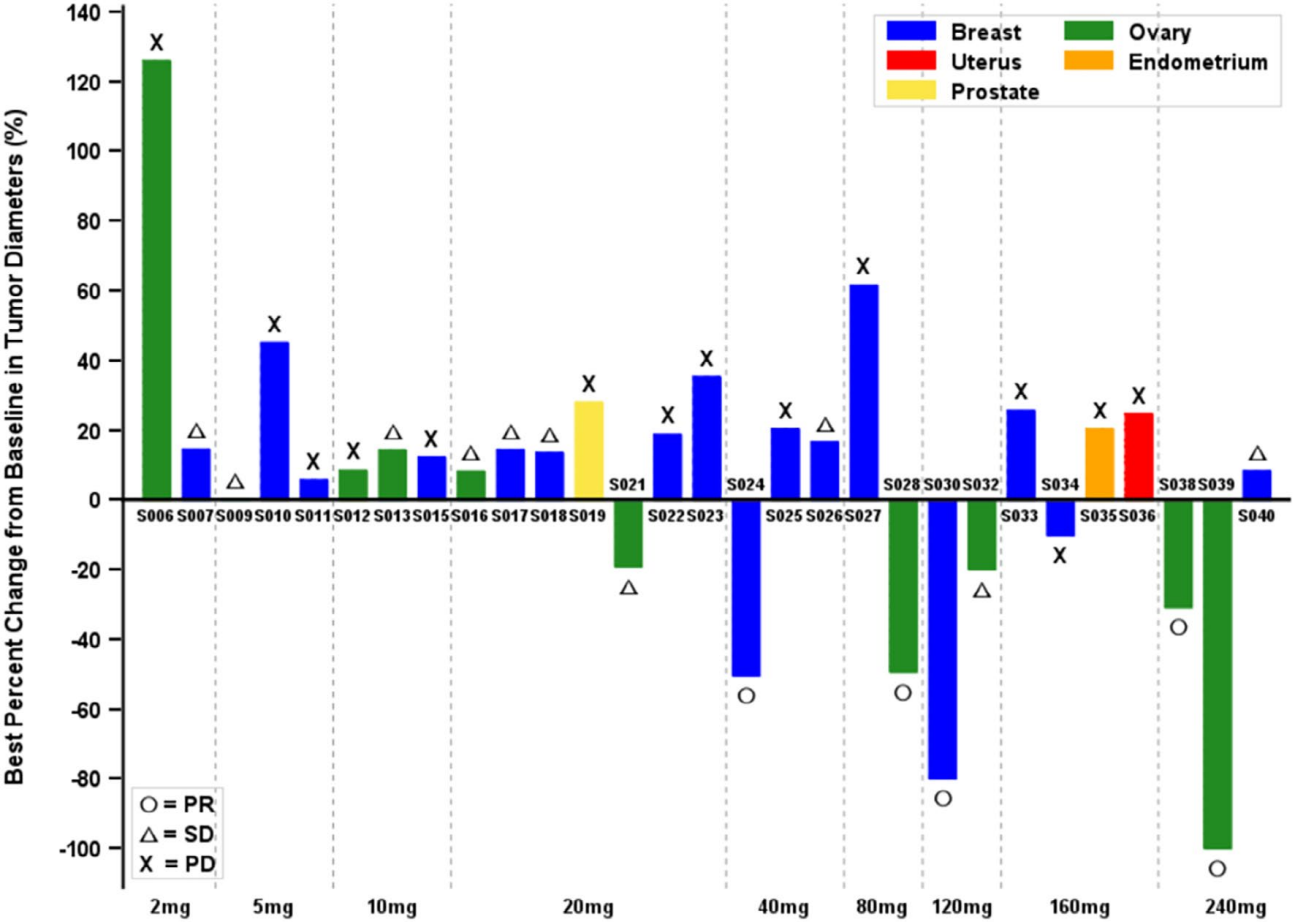
Best overall response with RECIST 1.1 tumor assessment in patients by dose.

Overall, 32 patients were treated. Three of these patients could not be evaluated for antitumor response: two withdrew consent before the first radiographic evaluation (Cohorts 7 and 8, 80 and 120 mg/d, respectively); one discontinued due to treatment delay caused by an adverse event that did not recover (Cohort 1, 2 mg/d). In the 29 evaluable patients, stable disease (SD) was observed from 2 mg/d and tumor shrinkage was observed from 20 mg/d. A dose–response relationship was observed based on the frequency of objective response and tumor volume reduction (%) (Figure 3, Table S1).

Among 16 breast cancer patients, 2 partial responses (PR) were observed: one in a TNBC patient with one prior palliative chemotherapy and the other in an HR+ patient with two prior palliative chemotherapies. These two patients received no prior platinum treatment. Among 12 ovarian cancer patients, 2 PR and 3 SD were observed in 9 patients with resistance to prior platinum regimens, while 2 SD were observed in 2 platinum‐sensitive patients. (Table S1).

**FIGURE 4:**
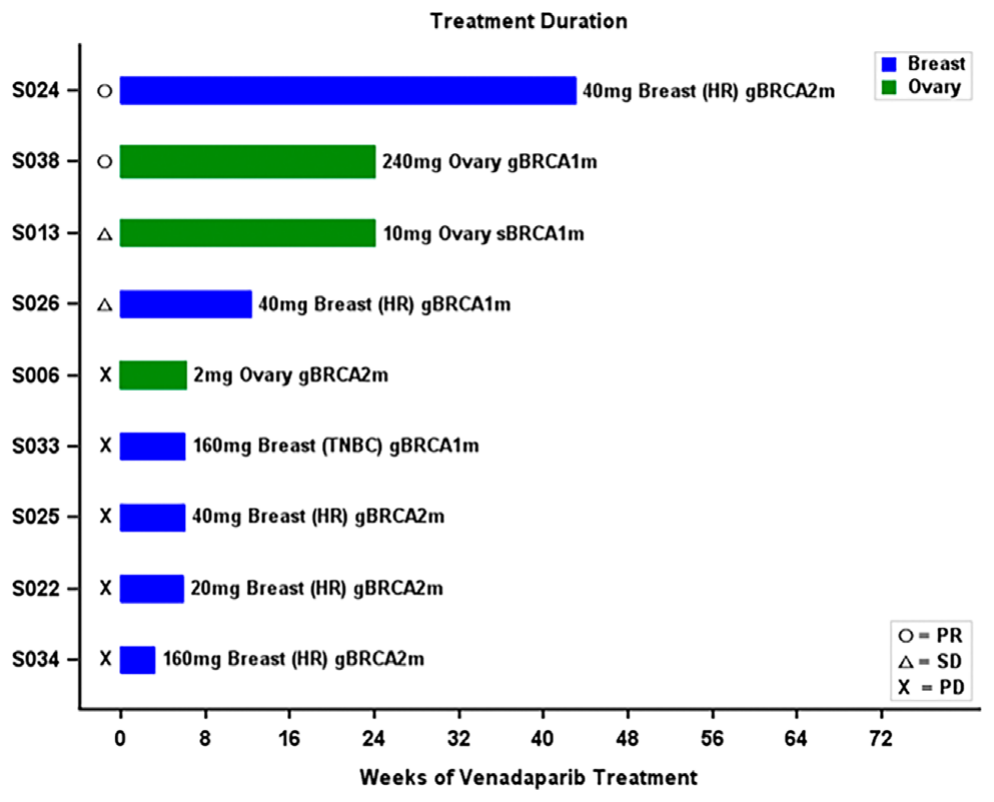
Swimmer diagram: Best overall response with RECIST 1.1 tumor assessment in patients with BRCA mutation by dose and cancer type (*N* = 9).

Among 9 patients with pathogenic BRCA mutations, the median (range) duration of treatment was approximately 6 (3–43) weeks, with 2 SD and 2 PR. A breast cancer patient (patient 024) with 2 prior hormone therapies followed by 2 palliative chemotherapies (no prior platinum therapy) in the 40 mg/d cohort showed PR (−50.4% maximum reduction) and was treated for 43 weeks. One ovarian cancer patient (patient 038) with platinum resistance in the 240 mg/d cohort showed PR (−30.9% maximum reduction) and was treated for 24 weeks. One ovarian cancer patient (patient 013) with platinum resistance and 6 prior palliative chemotherapies in the 10 mg/d cohort showed SD and was treated for 24 weeks. The median number (range) of prior palliative chemotherapies in the BRCA mutation group was 4 (1–6) (Figure 4, Table S1).

Amon 18 patients with wild‐type BRCA, the median (range) duration of treatment was around 9 (2–68) weeks; with 6 patients experiencing SD and 3 patients experiencing PR. Two ovarian cancer patients with PR were confirmed to have no other HRR mutation through retrospective circulating tumor DNA (ctDNA) analysis (Guardant OMNI panel). One breast cancer patient (patient 030), who was confirmed to have no HRR mutation through retrospective tissue NGS (Myriad myChoice HRD Plus CDx Test) with 1 prior palliative chemotherapy in the 120 mg/d cohort showed PR and was treated for approximately 24 weeks. (Table S1).

## 4 Discussion

Venadaparib is a novel PARP inhibitor with a potent PARP‐trapping effect [21]. This phase 1 trial demonstrated that venadaparib has an acceptable safety profile with a wide therapeutic window, ranging from the lowest to the highest dose tested. Its favorable pharmacokinetic and pharmacodynamic characteristics support once‐daily dosing.

During dose escalation, no MTD was identified in patients treated with venadaparib doses up to 240 mg once daily. Adverse events related to myelosuppression were reversible and primarily managed with drug interruptions or medical intervention. The side effects associated with venadaparib, such as anemia, neutropenia, nausea, vomiting, and fatigue are commonly observed with other PARP inhibitors. Compared to other PARP inhibitors in advanced ovarian cancer, venadaparib showed an improved gastrointestinal safety profile. In advanced ovarian cancer with ≥ 2 prior systemic treatments, approximately 76% and 40% of patients treated with olaparib experienced nausea and vomiting, respectively [33], while the corresponding rate for venadaparib were 38% and 16%, respectively. The frequency of hematologic adverse event was similar between venadaparib and olaparib: anemia (59% vs. 46%), neutropenia (28% vs. 24%), and thrombocytopenia (17% vs. 6%). Overall, venadaparib was well tolerated.

Venadaparib demonstrated favorable pharmacokinetic (PK) properties, including good oral bioavailability, rapid absorption, and dose‐proportional increases in total exposure (AUC) over a wide dose range (2–160 mg/d). Steady state was reached approximately 2 weeks after initiation of daily dosing. Linear urinary elimination kinetics were observed with daily dosing. At 160 mg/d, the half‐life (t_1/2_) was approximately 8.7 h upon multiple dosing.

In pharmacodynamic (PD) testing, venadaparib demonstrated PARP inhibition in PBMCs across a wide range of doses. For doses at and above 10 mg/d, PARP activity was consistently inhibited. These results suggest that effective PARP inhibition could be achieved at lower dose levels. Although the PBMC assay used was not PARP family‐specific, as peripheral blood cells are known to express both mono (ADP‐ribosyl) transferases and several PARP family enzymes, the PARP inhibition by 10 and 20 mg/d of venadaparib in tumor tissue lysates supports effective PARP inhibition. The PARP inhibition from lower dose levels together with improved gastrointestinal toxicity profile, suggests that low‐dose venadaparib may provide better compliance while maintaining efficacy in combination with chemotherapy, most of which have gastrointestinal side effects to a greater or lesser extent.

A signal of clinical benefit was observed in patients with BRCA mutations treated with venadaparib. Additionally, BRCA wild‐type patients also benefited from venadaparib, similar to observations in some previous PARP inhibitor studies. In the pre‐clinical development, venadaparib showed anti‐tumor efficacy; an IC50 of less than 10 nM in HR‐deficient ovarian cancer cell lines OVCAR‐3 and TOV‐21G was observed [21]. Even in the wild‐type BRCA1/2 cell line OVCAR4, the IC50 was 23 nM. In the OV_062 patient‐derived xenograft model without BRCA1 mutations but with MRE11A mutations and a high HRD score, venadaparib demonstrated a statistically significant tumor growth inhibitory effect in comparison with the control group and was superior to olaparib (100 mg/kg) [34]. HRD mutations were analyzed retrospectively using archival samples in all 5 patients with PR, and no HRD mutation was found in 2 ovarian cancer patients and 1 breast cancer patient (Table S1). We could not find a clear explanation for the contribution of non‐BRCA HRD in the response to venadaparib accordingly. This finding underscores the limitations of available assays to reliably detect specific HRD beyond BRCA mutations, despite the promising outcome of PARP inhibitors in the HRD patient population [33, 35].

Clinical benefit, defined as a radiologic or tumor marker response or stable disease for four or more months, was observed from as low as 20 mg/d of BRCA‐mutant group and 10 mg/d in the BRCA wild‐type group, where effective PARP inhibition of 90% or higher occurred. This is consistent with the results of olaparib, where clinical benefit started from 100 mg twice daily (BID), a dose higher than that required for effective PARP inhibition (> 60 mg BID of olaparib). In the current study, stable disease was observed from 2 mg/d given 2 of every 3 weeks, tumor shrinkage was observed from 20 mg/d, and partial response (PR) was observed form 40 mg/d. Although the minimum reproducibly active dose (MRAD) [36] was confirmed at 120 mg/d or higher, lower dose levels from 10 mg/d showed efficacy signals, supported by a strong pharmacodynamic profile of PARP inhibition. Considering the trend of dose–response in tumor volume reduction, the pharmacodynamic signal at lower dose levels, the absence of DLTs throughout the study, and PK linearity up to 160 mg/d, the recommended phase 2 dose (RP2D) was defined as 160 mg/d.

This study has limitations. First, diversity of cancer types and prior treatment exposure limit the interpretation of the results. The heavily pre‐treated patient population, particularly at lower dose levels (median [range] prior chemotherapy regimen of 5 [2, 3, 4, 5, 6, 7, 8, 9, 10, 11, 12] in the 2 ~ 20 mg/d groups and 2 [0–8] in the 40 ~ 240 mg/d groups), combined with tumor shrinkage observed from 20 mg/d and robust pharmacodynamic PARP inhibition from 10 mg/d, suggest the limitation of the classic 3 + 3 study design without backfill expansion at lower dose levels. Backfill or continuous dose expansion below MTD level might allow a more robust estimation of dose‐effect within sub‐group of cancer type and prior treatment exposure [36]. In addition, HRD status including BRCA could not be assessed in all patients recruited. Recruiting only patients with tumor or blood samples for retrospective centralized HRD testing could have provided a clear understanding of the biomarker‐response relationship of venadaparib treatment. Therefore, additional biomarker analysis, including intensive proteomic and genomic analyses of tumor tissue is necessary. These factors will be evaluated in the ongoing phase 1b/2a basket trial with multiple indication cohorts, enrolling breast, ovarian, and pancreatic cancers with HRD mutations (NCT04174716).

The wide therapeutic window, coupled with target engagement of PARP inhibition from low dose levels, supports the clinical development of venadaparib not only as monotherapy but also in combination with various anti‐cancer agents. Given its strong PARP inhibition and trapping potency, cytotoxic agents such as topoisomerase I inhibitor, platinum‐based agents, and alkylating agents, as well as immune check point inhibitors, may provide significant additive or synergistic efficacy when combined with PARP inhibitors [37, 38, 39]. Among chemotherapies, topoisomerase I inhibitors as well as alkylating agent are well known for their synergistic effect when combined with PARP inhibitors but their clinical application has not always been successful [39]. Irinotecan combined with existing PARP inhibitors for colorectal cancer and osteosarcoma failed mostly due to overlapping hematologic toxicity [40, 41, 42]. In the ongoing combination trial of venadaparib and irinotecan for advanced gastric cancer, venadaparib 20 mg/d (Day 1–7) and irinotecan 100 mg/m^2^ (Day 1) of a 14‐day cycle was determined as MTD, in which dose level, objective response greater than the current standard was reported [43]. Phase IIa part of dose optimization is ongoing. Further clinical development in the later‐line gastric cancer as well as small cell lung cancer and pancreatic cancer are under consideration with priority.

## 5 Conclusions

In conclusion, this study demonstrates the safety of single‐agent venadaparib for the treatment of patients with and without BRCA1/2 mutations in gynecologic cancers. Venadaparib has a tolerable safety profile and potential efficacy, and its PK/PD properties support once‐daily dosing.

## Author Contributions

**Sung‐Bae Kim:** data curation (lead), formal analysis (lead), investigation (lead), methodology (supporting), writing – original draft (supporting), writing – review and editing (supporting). **Kyun‐Seop Bae:** conceptualization (supporting), data curation (supporting), formal analysis (supporting), investigation (supporting), methodology (supporting), validation (supporting), writing – original draft (supporting), writing – review and editing (supporting). **Jae Lyun Lee:** formal analysis (supporting), investigation (supporting), writing – original draft (supporting), writing – review and editing (supporting). **Won Sik Lee:** data curation (supporting), formal analysis (supporting), funding acquisition (supporting), investigation (supporting), project administration (supporting), writing – original draft (supporting), writing – review and editing (supporting). **Chan‐Young Ock:** data curation (supporting), formal analysis (supporting), investigation (supporting), methodology (supporting), supervision (supporting), writing – original draft (supporting), writing – review and editing (supporting). **Myong‐Jae Lee:** conceptualization (lead), formal analysis (lead), investigation (supporting), methodology (lead), project administration (supporting), software (lead), validation (supporting), visualization (lead), writing – original draft (lead), writing – review and editing (supporting). **Jeongsook Bang:** data curation (supporting), formal analysis (lead), software (lead), visualization (lead), writing – original draft (supporting), writing – review and editing (supporting). **Min Ju Hong:** data curation (supporting), investigation (supporting), project administration (lead), writing – original draft (supporting), writing – review and editing (supporting). **Eun‐Jihn Roh:** formal analysis (supporting), writing – original draft (supporting), writing – review and editing (supporting). **Kyoung Soo Ha:** formal analysis (lead), visualization (lead), writing – original draft (supporting), writing – review and editing (lead). **Jong‐Ha Lim:** formal analysis (lead), methodology (supporting), validation (lead), writing – review and editing (supporting). **Yong‐Man Kim:** data curation (lead), formal analysis (supporting), investigation (lead), methodology (supporting), supervision (lead), writing – original draft (supporting), writing – review and editing (supporting).

## Ethics Statement

The study was approved by the Institutional Review Board, Asan Medical Center, Seoul, Republic of Korea and written informed consent was obtained from participants. This study was done in accordance with the Declaration of Helsinki and the Good Clinical Practice guidelines developed by the International Conference on Harmonization of Technical Requirements for Registration of Pharmaceuticals for Human Use. ClinicalTrials.gov ID is NCT03317743.

## Conflicts of Interest

S.B. Kim is a consultant on the advisory boards of Novartis, AstraZeneca, Lilly, Dae Hwa Pharmaceutical Co. Ltd., ISU Abxis, and Daiichi‐Sankyo, and has received research funding from Novartis, Sanofi‐Aventis, and DongKook Pharm Co., and owns stock in Genopeaks and NeogeneTC. W.S. Lee, M Lee, J. Bang, and M.J. Hong are employees of the sponsor. J.H. Lim is an employee of Ildong Pharmaceutical Co. Ltd. E.J. Roh and K.S. Ha are employees of Idience Inc. J.L Lee is a consultant on the advisory boards of Amgen, Astellas Korea, AstraZeneca Korea, BMS Korea, Merck, MSD Korea, and GI‐Innovation, and has received research funding from Ipsen Korea, Amgen, Pfizer, Novartis, BMS, MSD, Merck, AstraZeneca, Exelixis, SeaGen and Janssen. C.Y. Ock is employed by Lunit. K.S. Bae and Y.M. Kim declare no conflicts of interest.

## Supporting information


Appendix S1.

## Data Availability

Data supporting the findings of this study are available from the corresponding author, Yong‐Man Kim, upon reasonable request.
