# Supplementary material for: First‐In‐Human Dose Finding Study of Venadaparib (IDX‐1197), a Potent and Selective PARP Inhibitor, in Patients With Advanced Solid Tumors
Source: Cancer Med. 2025 Feb 13;14(4):e70576. doi: 10.1002/cam4.70576 (PMC11822664; doi:10.1002/cam4.70576)
Supplement: Supplementary file 1 — Appendix S1. [file CAM4-14-e70576-s001.docx]

**Supplementary Appendix Table 1. Disease characteristics and efficacy profile**

| **Subject ID** | **Age** | **Cancer type (subtype)** | **Dose**  **(mg/d)** | **Platinum sensitivity (Prior platinum regimen, n)** | **Prior line of palliative chemotherapy, n** | **ECOG** | **Genetic analysis** | | | | **Best overall response** | **Tumor marker response** | **Treatment duration, days** |
| --- | --- | --- | --- | --- | --- | --- | --- | --- | --- | --- | --- | --- | --- |
|  |  |  |  |  |  |  | **Site record** | **Archival blood sample** | **Archival Tumor tissue**^c^ | **Adjudicated** |  |  |  |
| 002 | 53 | Ovary | 2 | R (2) | 3 | 0 | BRCAwt | NA |  | BRCAwt | NE | No | 53 |
| 006 | 67 | Ovary | 2 | R (4) | 5 | 1 | gBRCA2m | NA |  | gBRCA2m | PD | No | 43 |
| 007 | 63 | Breast (HER2) | 2 | Not treated | 4 | 0 | NA | NA |  | NA | SD | NA | 83 |
| 009 | 44 | Ovary | 5 | S (2) | 4 | 0 | NA | NA |  | NA | SD | No | 85 |
| 010 | 56 | Breast (HER2) | 5 | Not treated | 7 | 1 | NA | NA |  | NA | PD | NA | 42 |
| 011 | 45 | Breast (HR+) | 5 | Not treated | 8 | 1 | NA | NA |  | NA | PD | NA | 42 |
| 012 | 75 | Ovary | 10 | R (2) | 9 | 1 | BRCAwt | NA |  | BRCAwt | PD | NA | 42 |
| 013 | 66 | Ovary | 10 | R (4) | 6 | 0 | sBRCA1m | BRCAwt^a, d^ |  | sBRCA1m | SD | No | 168 |
| 015 | 55 | Breast (TNBC) | 10 | Not treated | 3 | 0 | NA | NA |  | NA | PD | NA | 42 |
| 016 | 69 | Ovary | 20 | S (1) | 6 | 1 | BRCAwt | NA |  | BRCAwt | SD | No | 76 |
| 017 | 55 | Breast (HER2) | 20 | Not treated | 7 | 1 | BRCAwt | NA |  | BRCAwt | SD | NA | 84 |
| 018 | 58 | Breast (TNBC) | 20 | R (1) | 5 | 0 | NA | BRCAwt |  | BRCAwt | SD | NA | 127 |
| 019 | 70 | Prostate | 20 | R (1) | 12 | 1 | NA | BRCAwt |  | BRCAwt | PD | NA | 13 |
| 021 | 46 | Ovary | 20 | R (3) | 5 | 1 | NA | BRCAwt |  | BRCAwt | SD | Yes | 163 |
| 022 | 54 | Breast (HR+) | 20 | S (2) | 4 | 1 | gBRCA2m | BRCA2m^a^ |  | gBRCA2m | PD | NA | 41 |
| 023 | 41 | Breast (HR+) | 20 | R (1) | 2 | 1 | BRCAwt | BRCAwt |  | BRCAwt | PD | NA | 42 |
| 024 | 47 | Breast (HR+) | 40 | Not treated | 2 | 1 | gBRCA2m | NA | BRCA2m^c^ | gBRCA2m | PR | NA | 301 |
| 025 | 31 | Breast (HR+) | 40 | Not treated | 1 | 1 | gBRCA2m | BRCA2m^a^ |  | gBRCA2m | PD | NA | 42 |
| 026 | 53 | Breast (HR+) | 40 | R (1) | 6 | 1 | sBRCA1m | BRCA1m^a^ |  | gBRCA1m | SD | NA | 86 |
| 027 | 54 | Breast (HER2) | 80 | R (1) | 8 | 1 | BRCAwt | BRCAwt |  | BRCAwt | PD | NA | 41 |
| 028 | 59 | Ovary | 80 | NE (1) | 0 | 1 | BRCAwt | HRDwt^b, e^ |  | HRDwt | PR | No | 169 |
| 029 | 44 | Uterus | 80 | R (1) | 5 | 1 | NA | BRCAwt |  | BRCAwt | NE | NA | 37 |
| 030 | 53 | Breast (TNBC) | 120 | Not treated | 1 | 1 | BRCAwt | BRCAwt | HRDwt^c, e^ | HRDwt | PR | NA | 170 |
| 031 | 53 | Ovary | 120 | R (1) | 2 | 1 | BRCAwt | BRCAwt |  | BRCAwt | NE | No | 26 |
| 032 | 60 | Ovary | 120 | R (2) | 2 | 1 | BRCAwt | BRCAwt |  | BRCAwt | SD | No | 327 |
| 033 | 56 | Breast (TNBC) | 160 | S (1) | 2 | 1 | gBRCA1m | BRCA1m^a^ |  | gBRCA1m | PD | NA | 42 |
| 034 | 36 | Breast (HR+) | 160 | Not treated | 4 | 1 | gBRCA2m | BRCA2m^a^ |  | gBRCA2m | PD | NA | 22 |
| 035 | 58 | Endometrium | 160 | R (3) | 4 | 1 | NA | BRCAwt |  | BRCAwt | PD | No | 43 |
| 036 | 46 | Uterus | 160 | R (1) | 4 | 1 | BRCAwt | BRCAwt |  | BRCAwt | PD | No | 47 |
| 038 | 50 | Ovary | 240 | R (2) | 2 | 1 | gBRCA1m | NA | BRCA1m^c^ | gBRCA1m | PR | No | 168 |
| 039 | 57 | Ovary | 240 | R (2) | 5 | 1 | BRCAwt | HRDwt^b,e^ |  | HRDwt | PR | No | 476 |
| 040 | 59 | Breast (HR+) | 240 | Not treated | 2 | 1 | BRCAwt | BRCAwt |  | BRCAwt | SD | NA | 83 |

NA: not available, NE: not evaluable

^a^PBMC NGS of BRCA gene, Axen BRCA1/2 Cancer panel, Macrogen, Seoul, South Korea

^b^Plasma ctDNA analysis of HRR gene, Guardant OMNI panel, Guardant Health, Redwood, US

^c^Myriad myChoice® HRD Plus CDx Test, Myriad Genetics Salt Lake City, US

^d^wt: BRCA gene wild type

^e^wt: HRR wild type

**Supplementary Appendix Table 2. Noncompartmental analysis result of venadaparib by dosing group (day 1)**

| **Cohort** | **n** | C_max_ (ng/mL) | T_max_ (h) | AUC_inf_ (h*ug/L) | AUC_last_ (h*ug/L) | V_z_ (L) | T_1/2β_ (h) |
| --- | --- | --- | --- | --- | --- | --- | --- |
| 1 | 3 | 84.50±54.00  (63.9%)  [33.40-141.00] | 1.92  (1.03-1.97) | 780.62±401.58  (51.4%)  [318.42-043.92] | 593.00±274.64  (46.3%)  [291.48-828.86] | 47.20±13.15  (27.8%)  [34.62-60.85] | 11.64±4.54  (39.0%)  [6.72-15.66] |
| 2 | 3 | 180.00±48.57  (27.0%)  [133.00-230.00] | 3.00  (0.55-4.00) | 1827.86±1666.87  (84.7%)  [33.40-141.00] | 1340.46±1006.85  (84.7%)  [33.40-141.00] | 57.08±26.96  (84.7%)  [33.40-141.00] | 10.52±3.54  (33.7%)  [6.97-14.06] |
| 3 | 3 | 204.17±173.02  (84.7%)  [33.40-141.00] | 3.00  (2.00-8.02) | 3649.06±4836.80  (132.5%)  [144.88-9167.47] | 2519.97±3026.42  (120.1%)  [127.22-5922.09] | 333.81±505.10  (151.3%)  [21.07-916.53] | 9.94±3.14  (31.6%)  [7.23-13.39] |
| 4 | 3 | 380.00±146.81  (38.6%)  [228.00-521.00] | 3.00  (2.08-4.00) | 3039.02±674.82  (22.2%)  [2368.04-3717.62] | 2754.42±727.50  (26.4%)  [1991.23-3440.00] | 80.18±39.12  (48.8%)  [55.73-125.29] | 7.91±2.11  (26.7%)  [6.25-10.28] |
| 5 | 3 | 1128.00±443.52  (39.3%)  [862.00-1640.00] | 2.93  (0.50-3.00) | 12875.06±5816.24  (45.5%)  [7832.01-19189.58] | 10232.09±3651.74  (35.7%)  [7292.97-14320.11] | 23.05±4.34  (18.8%)  [19.75-27.97] | 9.98±3.76  (37.7%)  [5.82-13.13] |
| 6 | 3 | 2043.33±1547.82  (75.7%)  [1110.00-3830.00] | 2.00  (2.00-3.00) | 15825.90±13925.93  (88.0%)  [7229.41-31893.03] | 14369.29±12181.96  (84.8%)  [6801.11-28421.88] | 37.48±21.36  (57.0%)  [13.19-53.37] | 6.87±0.36  (5.3%)  [6.64-7.29] |
| 7 | 3 | 3643.33±582.27  (16.0%)  [2980.00-4070.00] | 1.03  (1.00-3.00) | 31699.75±12751.32  (40.2%)  [17104.01-40676.73] | 27733.70±10788.09  (38.9%)  [15336.37-34987.13] | 35.61±20.31  (57.0%)  [23.61-59.06] | 8.29±0.47  (5.7%)  [7.81-8.75] |
| 8 | 3 | 2923.33±1179.25  (40.3%)  [2030.00-4260.00] | 2.00  (1.95-2.07) | 28572.20±24412.45  (85.4%)  [12537.00-56667.70] | 23020.92±16230.34  (70.5%)  [12129.81-41674.88] | 61.75±17.99  (29.1%)  [41.34-75.31] | 8.56±4.44  (51.9%)  [4.97-13.53] |
| 9 | 4 | 5295.00±628.68  (11.9%)  [4820.00-6220.00] | 2.00  (1.00-3.00) | 48298.24±7488.56  (15.5%)  [39493.27-55858.21] | 42174.71±5663.76  (13.4%)  [35508.61-48321.59] | 39.02±4.02  (10.3%)  [36.11-44.88] | 8.15±1.31  (16.1%)  [6.18-8.88] |
| 10 | 3 | 4243.33±1097.38  (25.9%)  [2980.00-4960.00] | 2.03  (1.95-3.87) | 37514.17±11745.33  (31.3%)  [23968.05-44861.59] | 35064.82±10936.96  (31.2%)  [22440.89-41684.26] | 58.25±20.01  (34.4%)  [45.93-81.34] | 5.86±0.27  (4.5%)  [5.63-6.15] |

*Data are summarized as arithmetic mean ± standard deviation except for Tmax, for which median (minimum-maximum).

**Supplementary Appendix Table 3. Noncompartmental analysis result of venadaparib by dosing group (day 14)**

| **Cohort** | **n** | C_max,ss_ (ng/mL) | T_max,ss_ (h) | AUC_inf,ss_ (h*ug/L) | AUC_last,ss_ (h*ug/L) | V_d,ss_ (L) | T_1/2β_ (h) | AR^*^ |
| --- | --- | --- | --- | --- | --- | --- | --- | --- |
| 1 | 3 | 164.83±158.82  (96.4%)  [55.40-347.00] | 1.95  (0.50-3.00) | 1736.03±1605.80  (92.5%)  [457.71-3538.38] | 1364.99±1236.83  (90.6%)  [418.50-2764.45] | 26.55±15.98  (60.2%)  [9.51-41.19] | 10.12±3.11  (30.8%)  [6.53-12.16] | 2.05±1.11  (54.2%)  (1.38-3.38) |
| 2 | 3 | 274.27±162.91  (59.4%)  [90.80-402.00] | 2.92  (2.00-3.00) | 3471.41±2661.23  (76.7%) [854.73-6175.06] | 2104.75±1410.01  (67.0%) [709.76-3529.30] | 49.88±26.44  (53.0%)  [25.23-77.80] | 17.56±7.23  (41.1%)  [9.22-21.87] | 1.67±0.84  (50.6%)  (0.98-2.61) |
| 3 | 3 | 484.00±194.58  (40.2%)  [269.00-648.00] | 1.00  (1.00-6.00) | 7870.90±9667.28  (122.8%) [1452.44-18989.55] | 4925.17±5146.81  (104.5%) [1287.46-10814.03] | 47.43±33.75  (71.2%)  [13.09-80.56] | 12.01±4.70  (39.1%)  [8.11-17.22] | 7.90±11.40  (144.0%)  (0.85-21.00) |
| 4 | 3 | 462.00±198.65  (43.0%) [240.00-623.00] | 3.00  (2.08-4.00) | 4133.27±1804.89  (43.7%) [2714.79-6164.85] | 3664.78±1671.01  (45.6%) [2268.75-5516.31] | 68.11±35.43  (52.0%)  [34.78-105.328 | 8.39±1.33  (15.8%)  [7.43-9.91] | 1.29±0.27  (20.9%)  (1.13-1.60) |
| 5 | 3 | 1372.33±691.19  (50.4%) [867.00-2160.00] | 3.00  (2.00-3.00) | 19641.86±12129.06  (61.8%) [9377.20-33025.96] | 14356.35±8586.68  (59.8%) [7554.53-24004.86] | 23.71±11.37  (48.0%) [11.24-33.51] | 12.95±2.11  (16.3%) [10.89-15.11] | 1.33±0.32  (24.4%)  (1.04-1.68) |
| 6 | 3 | 2043.33±1547.82  (75.7%)  [1110.00-3830.00] | 2.00  (2.00-4.00) | 20437.84±21732.92  (106.3%) [4443.15-45181.74] | 15650.44±15761.44  (100.7%) [4158.86-33618.37] | 50.82±32.01  (63.0%)  [15.65-78.27] | 10.05±3.49  (34.7%)  [6.03-12.16] | 1.02±0.43  (42.6%)  (0.53-1.35) |
| 7 | 3 | 3220.00±3410.00  (5.9%)  [3030.00-3410.00] | 0.50  (0.50-3.00) | 35855.66±11046.67  (30.8%) [28487.65-48557.05] | 29755.39±5857.45  (19.7%) [25647.39-36462.67] | 29.77±1.44  (4.8%) [28.10-30.63] | 9.16±2.33  (25.4%) [7.54-11.82] | 1.18±0.44  (37.3%)  (0.83-1.67) |
| 8 | 3 | 3650.00±3170.05  (86.9%)  [1370.00-7270.00] | 2.08  (2.00-3.00) | 43329.91±51306.71  (118.4%) [9826.55-102396.16] | 34807.85±37475.34  (107.7%) [9506.28-77860.52] | 50.97±33.01  (64.8%) [19.83-85.58] | 7.15±3.96  (55.4%) [4.86-11.73] | 1.26±0.56  (44.2%)  (0.78-1.87) |
| 9 | 4 | 5860.00±2493.69  (42.6%)  [3610.00-8190.00] | 1.50  (1.00-3.02) | 64897.81±24747.53  (38.1%) [35388.15-95105.59] | 54295.35±20528.36  (37.8%) [31921.97-80064.80] | 37.11±12.87  (34.7%) [21.26-49.44] | 9.44±1.74  (18.4%) [7.58-11.70] | 1.28±0.43  (33.4%)  (0.90-1.78) |
| 10 | 3 | 4650.00±1574.20  (33.9%)  [2840.00-5700.00] | 3.05  (1.97-4.00) | 57242.36±29434.12  (51.4%) [25260.40-83194.93] | 51458.84±25578.53  (49.7%) [23517.49-73719.38] | 50.00±28.79  (57.6%) [28.90-82.80] | 6.66±0.54  (8.1%) [6.04-7.00] | 1.40±0.36  (25.7%)  (1.05-1.77) |

*Data are summarized as arithmetic mean ± standard deviation except for Tmax, for which median (minimum-maximum).

* Day 14 AUC_last_/Day 1 AUC_last_

**Supplementary Appendix Table 4. Venadaparib-Related Adverse Event by BRCA mutation**

| **Adverse Events** | BRCA mutation | | BRCA wild type | | Unknown | |
| --- | --- | --- | --- | --- | --- | --- |
|  | All grades | Grade 3-4 | All grades | Grade 3-4 | All grades | Grade 3-4 |
| **Subjects with any AEs** | 8 (89%) | 4 (44%) | 18 (100%) | 12 (67%) | 5 (100%) | 2 (40%) |
| **Blood and lymphatic system disorders** | | | | | | |
| Anaemia | 5 (56%) | 4 (44%) | 12 (67%) | 10 (56%) | 2 (40%) | 2 (40%) |
| Neutrophil count decreased | 3 (33%) | 1 (11%) | 6 (33%) | 5 (28%) |  |  |
| Platelet count decreased |  |  | 2 (11%) | 2 (11%) |  |  |
| **Gastrointestinal disorders** | | | | | | |
| Nausea | 6 (67%) |  | 5 (28%) |  | 1 (20%) |  |
| Vomiting | 2 (22%) |  | 2 (11%) |  | 1 (20%) |  |
| Dyspepsia | 2 (22%) |  | 2 (11%) |  |  |  |
| Abdominal pain | 1 (11%) |  | 1 (6%) |  |  |  |
| **Metabolism and nutrition disorders** | | | | | | |
| Decreased appetite | 2 (22%) | 1 (11%) | 2 (11%) |  | 1 (20%) |  |
| Hypoalbuminaemia | 1 (11%) |  | 1 (6%) | 1 (6%) |  |  |
| Hypertriglyceridaemia |  |  |  |  | 2 (40%) |  |
| **General disorders and administration site conditions** | | | | | | |
| Fatigue | 2 (22%) |  | 3 (17%) |  |  |  |
| Asthenia | 1 (11%) |  |  |  | 1 (20%) | 1 (20%) |
| Influenza like illness | 2 (22%) |  |  |  |  |  |
| **Nervous system disorders** | | | | | | |
| Headache | 1 (11%) |  | 1 (6%) |  | 1 (20%) |  |
| **Infections and infestations** | | | | | | |
| Urinary tract infection |  |  | 1 (6%) |  | 1 (20%) |  |
